# Enrichment-Free Rapid Detection of Phthalates in Chinese Liquor with Electrochemical Impedance Spectroscopy

**DOI:** 10.3390/s20030901

**Published:** 2020-02-07

**Authors:** Xinyue Jiang, Yuqun Xie, Duanji Wan, Fuping Zheng, Jun Wang

**Affiliations:** 1 School of Bioengineering and Food Science, Hubei University of Technology, Wuhan 430068, Hubei, China; xinyuejiang@hbut.edu.cn; 2 School of Civil Engineering, Architecture and Environment, Hubei University of Technology, Wuhan 430068, Hubei, China; wanduanji@163.com; 3 Beijing Advanced Innovation Center for Food Nutrition and Human Health, Beijing Technology and Business University, Beijing 100048, China; zhengfp@th.btbu.edu.cn

**Keywords:** in-situ preconcentration, phthalates, graphene, electrochemical impedance spectroscopy

## Abstract

A non-invasive real-time detection technique for phthalates in Chinese liquor is proposed in this paper. This method is based on the measurement of Faradaic impedance in the presence of a redox probe, [Fe(CN)_6_]^3−/4−^, upon the absorption of phthalates to the graphene electrode surface. This absorption activity is according to the π–π stacking interactions between phthalates and the graphene working electrode which allows direct sampling and analyte preconcentration. The absorption of phthalates retards the interfacial electron-transfer kinetics and increases the charge-transfer resistance (R_ct_). Numerical values of R_ct_ were extracted from a simulation of electrochemical impedance spectroscopy (EIS) spectra with the corresponding equivalent circuit. Cathodic polarization was employed prior to EIS measurements to effectively eliminate the metal ion interference. The results yielded a detection limit of 0.024 ng/L for diethyl phthalate (DEP) with a linear range from 2.22 ng to 1.11 µg. These results indicate a possibility of developing a household sensor for phthalate determination in Chinese liquor.

## 1. Introduction

Since the outbreak of phthalates esters (PAEs) in Chinese liquor, plasticizers have been detected in beverages, infant drinks, soy sauce, vinegar, and other condiments in succession [1,2]. The potential environmental and human health impacts caused by PAEs are spreaded into our daily life [3,4,5,6]. PAEs are very widely employed as additives for polymers in plastic, particularly in polyvinyl chloride (PVC) and polyethylene terephthalate (PET), which are widely applied in rubber, cellulose, and in the production of styrene as well [7]. PAEs help to improve the flexibility, transparency, and durability of articles manufactured with polymeric matrixes due to its low production cost. Unfortunately, some illegal food producers take advantage of the above feature of PAEs, adding PAEs instead of palm oil into food products to sustain the desired texture, resulting in severe harm to consumers [8,9]. Besides intentional addition, PAEs migrate from plastic packaging to the articles of consumption. This is attributed to the fact that the plasticizer molecules are only physically attached to polymer chains rather than via primary bonding [10,11,12].

Currently, some of the most common and efficient approaches for detecting PAEs are gas chromatography–mass spectroscopy (GC/MS) and liquid chromatography–mass spectrometry (LC/MS) [13,14]. The aforementioned methods provide high resolution and sensitivity to determine multiple PAEs simultaneously. However, they are destructive techniques which require multi-step sample pretreatment and relatively high cost. Recently, some researchers have explored the application of near-infrared (NIR) spectroscopy to detect PAEs in red tea beverage. The results showed a lack of sensitivity—the detection limit was about 15.8 mg /L [15]. Zhang et al. developed an enzyme-linked immunosorbent assay (ELISA) approach which performed the analysis with elevated sensitivity and detection limit compare to NIR. However, ELISA involves complicated procedures such as antibody production, purification, and derivatizations, which hinder its application [16]. Molecularly imprinted polymers (MIP)-based electrochemical sensors have drawn prominent attention, and a di-n-butyl phthalate (DBP) electrochemical sensor was constructed applying magnetic graphene oxide and gold nanoparticles as a molecular recognition element. It showed a 222.6 ng/ L detection limit with excellent repeatability (relative standard deviation (RSD), 2.5%) [17]. MIP also exhibits some notable drawbacks, such as the complicated preparation process, low binding capacity, and poor site accessibility [18]. An inter-digital sensor with multiple sensing gold electrodes fabricated on a silicon substrate using micro-electromechanical system (MEMS) device fabrication technology was reported in 2013 [19], and it provided a sensitive and rapid approach for detecting PAEs in water and juice samples with EIS. The results proved the applicability of EIS, however the reported MEMS method requires complicated working electrode fabrication. Table 1 illustrates the present state of PAEs analysis.

Although the analytical methods described above are suitable for the administration of manufacturing and quality control departments, they are helpless to dispel the concerns caused by PAEs in people’s daily life. Therefore, there is an urgent demand for the development of a facile, highly sensitive, low-cost PAEs analysis technique.

**Figure 1:**
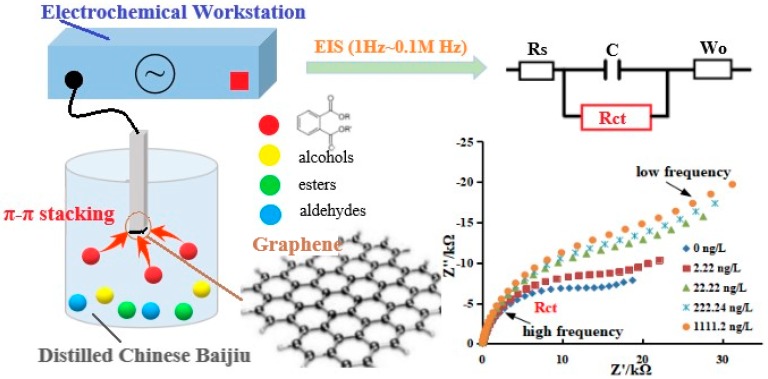
A schematic diagram illustrating the basic strategies of in situ preconcentration and detection of PAEs in Chinese liquor with electrochemical impedance spectroscopy.

The objective of the presented work is to demonstrate an enrichment-free detection approach for PAEs analysis in Chinese liquor with electrochemical impedance spectroscopy (EIS). A graphene working electrode was employed to accomplish the in-situ preconcentration of PAEs according to the π–π stacking interactions between graphene and benzene rings in PAEs molecules. The absorption of PAEs retards the interfacial electron-transfer kinetics and increases the charge-transfer resistance (R_ct_). Numerical values of R_ct_ were extracted from a simulation of EIS spectra with the corresponding equivalent circuit. Cathodic polarization was employed prior to EIS measurements to effectively eliminate the metal ion interference. Figure 1 outlines the basic strategies of the PAEs sensor based on the EIS measurements applying graphene as the working electrode. This work implicates the potential of developing a household device for PAEs determination.

## 2. Materials and Methods

### 2.1. Chemicals and Materials

All aqueous stock solutions were prepared with deionized water purified by a membrane device. Ethanol (absolute) and acetone were obtained from (Tianjin Fuchen Chemical Reagent Factory, Tianjin, China). Diethyl phthalate (DEP) was obtained from Macklin (Shanghai, China). Copper (II) chloride dihydrate, sodium chloride, and potassium ferricyanide were all obtained from (Sinopharm Chemical Reagent Co., Ltd., Shanghai, China). Graphene was obtained from (YURUI Chemical Co., Ltd., Shanghai, China). The purchased graphene was 1–5 layers, its purity was greater than 95%, its thickness distribution was from 1 to 1.77 nm, with diameters ranging from 0.5 to 5 µm. C18 solid-phase extraction cartridges were purchased from CNW Technologies (Shanghai, China).

### 2.2. Apparatus and Software

The apparatus consists of an electrochemical workstation (CorrTest, Wuhan, China) with a conventional three-electrode system, including a glassy carbon electrode as the working electrode, an Ag/AgCl electrode as the reference electrode, and a carbon rod as the auxiliary electrode. Other equipment included a KQ5200DE CNC ultrasonic cleaning machine (Shanghai, China); a DHG-9053A electric thermostatic blast drying oven (Kunshan, China); and a XB220A electronic analytical balance (Precisa). A GC7890B-MS5977B instrument from Agilent (Santa Clara, CA, USA) was used for chemical analysis. For analog equivalent circuit software, CorrTest and CSStudio were used.

### 2.3. Preparation of Graphene Electrode

The graphene working electrode was manufactured by dropping a mixed solution containing tetrahydrofuran and graphene onto a clean glassy carbon electrode to form a thin conductive film [28,29]. Prior to each manufacturing process, the glassy carbon electrode was polished with aluminum powder and cleaned with an ultrasonic cleaner. The graphene was dispersed in tetrahydrofuran in an ultrasonic cleaner for 30 min, then 5 µL of the dispersed graphene solution was dropped onto a cleaned glassy carbon electrode. Each electrochemical measurement was carried out after the tetrahydrofuran was completely volatilized in the air.

### 2.4. Electrochemical Measurements

The EIS measurements were taken with a three-electrode system. The working electrode was the graphene electrode, the Ag/AgCl electrode was used as the reference electrode, and the graphite carbon rod was the auxiliary electrode. A 10 mV AC amplitude was employed with a frequency range 1 Hz–0.1 M Hz. All the testing solutions contained 0.01 M potassium ferricyanide and 0.5 M sodium chloride as supporting electrolytes.

### 2.5. Standard Solutions Preparation

The stock solution was made by dissolving DEP in 50% ethanol. A serial dilution was followed up to obtain 0.01 nM, 0.1 nM, 1 nM, and 5 nM DEP solutions in total volumes of 10 mL containing 10 mM potassium ferrocyanide and 0.5 M potassium chloride as electrolyte.

### 2.6. Interference Elimination

Cathodic polarization was employed to eliminate the metal ions interference in the liquor samples. Graphite carbon rods were used as working and auxiliary electrodes, the Ag/AgCl electrode was used as the electrical reference electrode, the polarized potential was −0.1 V vs. Ag/AgCl. An artificial liquor sample was made from 60% ethanol solution with 0.5 μM DEP and 0.5 mM CuCl_2_ addition. This artificial liquor solution underwent cathodic polarization with an appointed duration prior to EIS measurement. The efficiency of copper metal ion elimination was determined by the ratio of the measured DEP concentration according to the standard curve to the original DEP concentration (0.5 μM).

### 2.7. Standard Addition

Concentrated DEP solution was spiked into 8 mL real liquor samples dropwise to perform the standard addition quantification. The final serial concentrations of DEP in liquor were 0, 5, and 10 μM with total volume of 9 mL. The above liquor solution underwent electrochemical polarization at −0.1 mV (vs. Ag/AgCl) to eliminate metal ion interference. After cathodic polarization, 1 mL 0.1 M potassium ferrocyanide in 0.5 M NaCl as supporting electrolyte was added for EIS measurement.

### 2.8. Method Validation with GC/MS

First, 10 mL of the Chinese liquor sample underwent solid-phase extraction with a C18 cartridge, which was rinsed with 12 mL 50% methanol. The sorbent was washed off by 20 mL water, the absorbed compounds were de-absorbed with 20 mL hexane, and then dried by nitrogen purge. The volume of extracts was adjusted to 10 mL for GC analysis. All samples were prepared in triplicate for repeatability tests. GC/MS experimental conditions were according to [19] in brief. External standard and standard addition were applied for the calibration curve preparation and PAEs determination in the real sample. A linear regression was employed for the calibration curve and was plotted by Excel software.

### 2.9. Quality Control and Quality Assurance

Quality assurance and quality control measurements were applied for all sample pretreatment and analytical procedures. Laboratory blanks (i.e., solvent and matrix) were treated equally as samples. Background variation was considered to be negligible during storage and analysis, when there was no significant difference was found (*t*-test, *p* < 0.05) between analyte concentrations in the analytical blanks. Limits of detection (LODs) were calculated based on the signals three times greater than the standard deviations of the average background signals of the blanks.

## 3. Results

### 3.1. Advantages of Graphene Electrode

**Figure 2:**
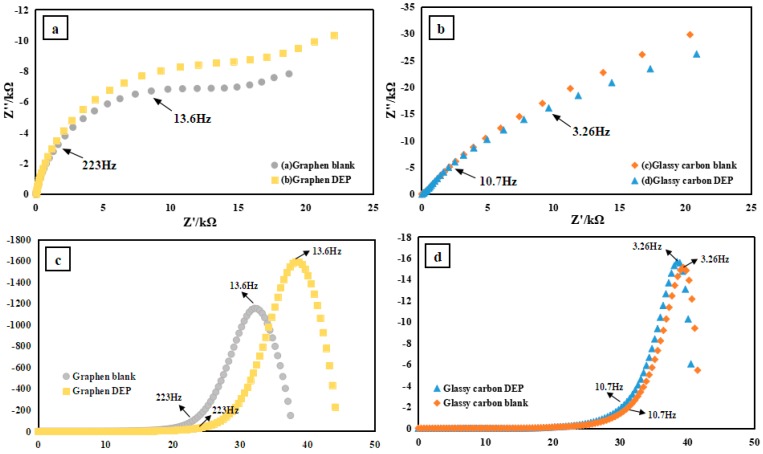
(**a**,**b**) EIS response comparisons of glassy carbon electrode and graphene electrode. The blank solution contained 0.5 M NaCl and 0.01 M K_3_[Fe(CN)_6_]. The DEP solution was prepared by adding 100 μM diethyl phthalate to the blank solution. EIS was carried under an open circuit voltage with an AC amplitude of 10 mV and a frequency range of 1 Hz–0.1 MHz. (**c**,**d**) Show (**a**,**b**) after deconvolution treatment, respectively.

Graphene-based materials have been extensively applied because their characteristic structure endows them with a large surface area, and because of the potential for establishing π–π stacking interactions due to graphene’s delocalized electrons, allowing them to be utilized as excellent sorbents [30,31,32]. Herein, a graphene working electrode was employed to accomplish the PAEs’ in-situ preconcentration according to the π–π stacking interactions between graphene and PAEs which consist mainly of one benzene ring and two aliphatic ester groups attached to the benzene ring in an ortho configuration. EIS was employed to characterize PAEs’ absorption on the working electrode. The complex impedance is displayed as the sum of the real (Z’) and imaginary (Z”) components. A typical shape of a Faradaic impedance spectrum presented in a Nyquist plot includes a semicircular region lying on the Z’ axis followed by a straight line. The semicircle portion, observed at high frequencies, corresponds to the electron-transfer-limited process, whereas the linear part is characteristic of the lower frequency range and represents the diffusion-limited electron transfer process. Figure 2a,b shows the Nyquist plots of graphene and glassy carbon electrode with and without PAEs addition, respectively. Figure 2b appeared superimposed at high frequency with a slight deviation at low frequency, indicating the poor PAEs absorption on the surface of the glassy carbon electrode and thus a nonsignificant charge-transfer-resistance alteration. A different feature appeared on the Nyquist curves of graphene with and without PAEs addition. Figure 2a shows a pair of semicircles with different diameters, which suggests that the effective absorption of PAEs on the graphene electrode led to an increased charge transfer resistance. To achieve better resolution, a deconvolution treatment was applied to Figure 2a,b, which resulted in Figure 2c,d. In Figure 2c, two clearly separated peaks start to deviate at 223 Hz, reaching a summit at 13.6 Hz. This can be compared to Figure 2d, where two peaks are superimposed together without significant resolution.

### 3.2. Standard Curve

**Figure 3:**
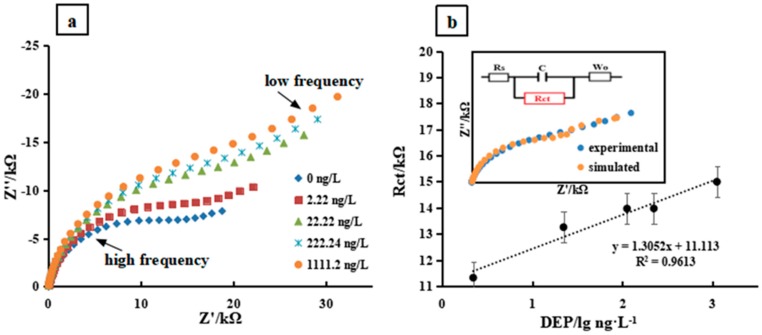
(**a**) Impedance spectrum of PAEs concentration gradient. The concentration of DEP was 0, 2.22, 22.22, 222.24, and 1111.2 ng/L, respectively. The measured solution contained 0.5 M NaCl and 0.01 M K_3_[Fe(CN)_6_]. EIS was measured under an open circuit voltage with an AC amplitude of 10 mV and a frequency range of 1 Hz–0.1 MHz. Each concentration was measured three times, and its mean value was plotted; (**b**) The R_ct_ values with respect to corresponding DEP concentrations. The inset shows a representative simulated Nyquist curve superposed with the experimental measurements according to corresponding equivalent circuits.

EIS measurements were carried out with a concentration series of DEP solutions from 0.01 to 5 nM on the graphene working electrode. From Figure 3a, at high frequency the diameters of the semicircle portion which represents interfacial electron-transfer resistance increased with increasing concentrations of DEP, implying that the DEP absorption on the surface of the graphene electrode was proportional to its concentration. Data pre-processing is critical at this point. A Nyquist plot simulation was carried out based on the equivalent circuit with CorrTest and CSStudio. Simulated curves were precisely fitted with experimental Nyquist plots at high frequency in which the vital features of EIS measurement dominated. The retarded electron transfer phenomena due to the DEP absorption was expressed at high frequency. At low frequency, a tiny deviation appeared, in which the mass transfer dominated. This part of the EIS information is irrelevant for the PAEs’ determination. Simulated curves remained the major electrochemical properties of PAEs on graphene electrode. Thus, the characteristic of Nyquist plots was translated into a set of circuit elements numerical values after curve fitting. Based on the equivalent circuits, the numerical values of R_ct_ were extracted to build a linear relationship between the electron-transfer resistance and logarithmic value of DEP. Concentrations of DEP were found ranging from 0.1 to 5 nM with a slope of 1.31 and a correlation coefficient of 0.9613 (Figure 3b) and a detection limit of 0.024 ng/L [33].

### 3.3. Elimination of Metal Ions Interference in the Chinese Liquor Sample

**Figure 4:**
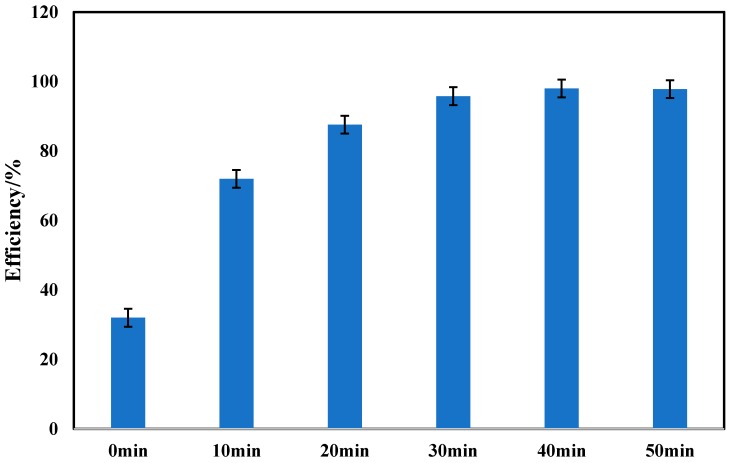
Metal ions elimination efficiency vs. cathodic polarization time. Graphite carbon rods were used as working and auxiliary electrode, Ag/AgCl electrode as the electrical reference electrode, polarized potential was −0.1 V vs. Ag/AgCl.

Chinese liquor is a distilled spirit made from mixed grains fermentation, which is usually stored in pottery containers after distillation for a certain period of time to obtain desired flavors. Therefore, notable metal ions migrate from the pottery jars to the liquor during the storage process [34,35,36]. The amount of metal ions in the liquor become problematic due to the similar hydrophobic features of the graphene working electrode and the metal ions. Metal ions adsorbed on the working electrode surface which give faulty signals during the EIS data collection for PAEs determination. Prior to EIS measurements, cathodic polarization with a graphite rod as working electrode at −0.1 V vs. Ag/AgCl was carried out to absorb or/and reduce metal ions on the surface of the graphite working electrode. Figure 4 presents the metal ion elimination efficiency vs. cathodic polarization duration. Without cathodic polarization pretreatment, only 30% of PAEs added into the artificial liquor were detected. The efficiency climbed up to 90% rapidly with increasing polarization time, and this reached a plateau after 30 min. This phenomenon was due to the low concentration of residual metal ions in the solution. The efficiency slightly increased to 98% until 40 min; the efficiency may infinitely approach 100% percent by continuing proceeding cathodic polarization long enough.

### 3.4. PAEs Quantification in Chinese Liquor

Standard addition was applied to determine DEP in real liquor samples in order to avoid the matrix effect. A 40 min cathodic polarization was carried out to eliminate the metal ion interference prior to EIS measurement. Detected DEP concentrations including standard deviations in real liquor are listed in Table 2. To validate the EIS measurements, GC/MS was employed to determine DEP in three liquor samples. Errors between these two sets of results were from +0.034 mg/L to −0.021 mg/L. The reason for these errors is that graphene electrode absorbs all kinds of PAEs besides DEP. As a major PAE, DEP coexists with other PAEs in liquor, and the EIS approach for PAEs analysis lacks specificity. This indicates that the presented method is suitable for total PAEs detection in liquor samples rather than for the detection of specific PAEs.

## 4. Conclusions

A method for the in-situ preconcentration and detection of PAEs in Chinese liquor with EIS is demonstrated. The elimination of metal ions interference prior to EIS measurement minimized the possibility of false positives. Compared to the GC/MS approach for PAEs detection, the demonstrated method offered a lower detection limit of 0.024 ng/L without a preconcentration step, and the entire PAEs analysis process was accomplished in few minutes. In terms of simplicity, speed, and low detection limits, the proposed method outperformed the techniques in the literature. We believe that these features may be incorporated into an inexpensive, rapid, and selective usable household device.

## Figures and Tables

**Table 1:** Summary of literature methods aimed at phthalates ester (PAEs) determination.

Analytical Technique	Matrix	Compounds	LOD ng/L	Sample Pretreatment	Reference
TLC	Glucose	DEP	2.4	Multi-step	[20]
Fluorescence	Waste water	DEP	18,440	None	[21]
GC	Water	DEP	3.7	Liquid phase extraction	[22]
(GC-MS/MS)	Chinese liquor	DEP	100	Liquid phase extraction	[23]
GC-MS	Alcoholic beverages	DEP	700,000	Centrifugation extraction	[7]
LC-DAD	Red wine	DEP	2000	Separation and elution	[7]
HPLC	Rainwater	DEP	200	In-tube SPME	[24]
Electrochemical Sensor	Chinese liquor	DINP	11,300	None	[25]
UHPLC-MS	Waste water	DBEP	6	SPEM	[26]
HPLC-MS	Beverage	9 PAEs	173	SPE	[27]

TLC: thin layer chromatography; GC: gas chromaograph; GC-MS/MS: gas chromatography tandem triple quadruple mass spectrometry; GC-MS: gas chromaographymass spectrometry; LC-DAD: liquid chromatography-diode array detector; HPLC: high-performance liquid chromatography; UHPLC-MS: ultra high performance liquid chromatography-mass spectrometry; HPLC-MS: High performance liquid chromatography-mass spectrometry. DINP: diisononyl phthalate; DBEP: dibutyl ethyl phthalate. LOD: limit of detection. SPEM: micro-dispersive solid-phase extraction; SPE: solid-phase extraction.

**Table 2:** The comparison of DEP analysis in real liquor samples with GC/MS and EIS methods.

Sample	EIS		GC/MS		Error (mg/L)
	DEP (mg/L)	Standard Deviation (*N* = 3)	DEP (mg/L)	Standard Deviation (*N* = 3)	
Xiaoqu liquor	0.149	0.021	0.135	0.012	+0.014
Daqu liquor	0.170	0.018	0.191	0.025	−0.021
Medicinal liquor	1.234	0.020	1.200	0.024	+0.034
